# Aged Persons Living with HIV and Nutritional Wellness: Analysis of 2013 South Africa-SAGE Well-Being of Older People Study (WOPS) Wave 2

**DOI:** 10.1155/2021/6635814

**Published:** 2021-06-23

**Authors:** Joseph Kojo Oduro, Kwaku Kissah-Korsah

**Affiliations:** Department of Population and Health, College of Humanities and Legal Studies, University of Cape Coast, Cape Coast, Ghana

## Abstract

**Introduction:**

This study sought to examine the nutritional wellness among aged persons living with HIV in Somkhele, South Africa.

**Methods:**

Data were extracted from the 2013 South Africa-SAGE Well-Being of Older People Study (WOPS) Wave 2. The study sampled 440 aged persons (50 years and above). The proportion of the aged persons with high nutritional wellness by key covariates was reported with chi-square and *p*-values (*p* < 0.05). Two-level binary logistic regression models were fitted.

**Results:**

Results show that there were more aged women (79.8%) than men (20.2%) and the younger old (50–64) dominated (65.7%) in the ages, among the respondents. A higher percent of the aged persons were widows (39.5%) and had no formal education (46.1%). Six in 10 aged persons were HIV infected (59.5%). Percentage distribution of men with high nutrition was higher (78.7%). Regression results show that having nutritional wellness was low for aged persons who were infected by HIV (OR = 0.74, 95% CI = 0.69, 1.26) when compared to those who were not infected. Regarding ages of respondents, having high nutritional wellness was higher for young old (65–74 years) (OR = 1.21, 95% CI = 0.65, 2.25) compared to younger old (50–64 years).

**Conclusion:**

This study suggests that age, education, source of drinking water, household source of income, and financial situation are important for nutritional wellness of aged persons who are HIV infected in South Africa. Moreover, having HIV infection is associated with low nutritional wellness. Being a young old (65–74 years) and receiving government grants and with better financial situation is associated with high nutritional wellness. Stakeholders and agencies who have interest in aged persons affected by HIV must understand the socioeconomic status in relation to their nutritional wellness. The results are of great importance to ageing policies, specifically in health and nutrition.

## 1. Introduction

Insight into the nutritional wellness and HIV-infected aged persons is imperatively a global public health concern. Nutritional wellness is extremely crucial to preserving a healthy life among the aged [1]. Notably, there is a biological, functional, and psychological decline and risk of disease in the process of ageing [2]. Ageing is associated with a decline in the body's capacity to respond to stress, and this makes the aged more prone to infectious diseases such as the human immunodeficiency virus (HIV) [3, 4]. In this regard, well nutritional therapy is a vital adjunct in the care of the aged living with HIV. Attaining and sustaining optimum diet can improve the immune function, decrease the occurrences of complications that are associated with HIV infection, attenuate the development of HIV infection, advance quality of life, and eventually reduce mortality associated with HIV among the aged [5, 6]. Thus, nutrition has an enormous bearing on the physical health and well-being of the aged.

Those aged 50 years and over account for 13% of global adult population living with HIV [7]. In sub-Saharan Africa, 60% of this cohort (50+) live with HIV. Thus, the population of the aged 50+ and living with HIV constitutes 16.1% in South Africa [7]. Over two decades into the epidemic, HIV is considered simply as beyond just medical issue, with implications well outside the traditional medical model of disease [5]. Effective responses to HIV among the aged are multisectoral and multifaceted. Amid the sectors, support for nutritional wellness should be given a key consideration in response to issues concerning the aged living with HIV [5].

HIV is known to negatively impact the nutritional health of an individual in three underpinning ways including the fact that it alters the body's metabolism, so that more energy and micronutrients are demanded and utilized [3]. Secondly, individuals with HIV often eat less food due to loss of appetite, mouth or throat sores, pain and nausea, side effects of medication, or worsening household poverty and livelihood security, and finally, it impairs the absorption of nutrients consumed on account of diarrhea and vomiting, damaged intestinal cells, and other effects of opportunistic infections [3].

Studies show that poverty, poor diet, unsafe food, and drinking water together with milk supplies were amid the sources of the all-pervading infectious diseases and early death [7, 8]. Although most of them age in place and live independently, these aged, especially those living with HIV, may be placed at high nutritional risk at some point in time of the ageing process when food-related activities are disordered [9]. The aged who are married and living independently are the least to experience low nutritional wellness. However, living alone increases the chance of not eating recurrently, predominantly among men, unless prospects to socialise and share meals are available [9].

Evidence on aged persons with HIV prevalence and nutritional wellness is limited across the sub-Saharan Africa. For instance, Gregório et al. [10] found HIV prevalence ranging between 5% and 13% among younger-old (50–64 years) in Kenya and South Africa. In Swaziland, HIV prevalence rate was between 13% and 7% in men and women among young-old (65–74 years), respectively. Some other studies have shown that nutritional wellness is associated with better quality of life [11–13]. Individuals with a higher score for observance to a Mediterranean-style diet [14, 15] and higher consumption of low-fat milk and yogurt [16] have lower odds of developing frailty, while a variety and quality of diets in aged persons are increased by factors, such as social resources, money, and help from friends and neighbours [17].

Despite the fact that extremely little insight is available in the application of theory and literature on HIV status and nutritional wellness among the aged, knowledge about the influence of diet patterns in relation to HIV status provides valuable insights into how society can promote healthy lifestyle to an ageing population by increasing aged person's food knowledge [18]. This study sought to examine HIV status and nutritional wellness among aged persons in Somkhele, South Africa.

Not astonishingly, worldwide natural science concepts have often included the theory of nutrition as an extremely vital part in their investigations. In its modest form, the classical theory of a balanced diet focuses on the assumption that the body should have a supply, composed of such molecular structure, that would compensate for their expense and loss from the metabolism, work, and growth for both the young and the old [19]. It is about shared belief on the ideal food and optimal balanced diet. It is based on a balanced approach for the assessment of diet [19]. The classical theory of a balanced diet best explains nutritional wellness of aged persons living with HIV [19].

Studies that have applied the classical theory of a balanced diet include the evidence that availability and accessibility of healthy food, social support, and socioeconomic status are significant for nutritional wellness [20]. Besides, Aikin and Apt [21] concluded that a good understanding of nutritional wellness improves individual health status and also prevents disease and promotes well-being for older and younger generations. Guralnik and Ferrucci [22] assert that until we recognize the intricacy of nutrition and scientifically monitor its consequences from the food setting to eating choices and its health effects, it will be difficult to control epidemic like obesity-related diseases risen in recent times. Notwithstanding this, the classical theory of a balanced diet is generally applied in the study of nutrition and health status for both the aged and young population.

**Figure 1:**
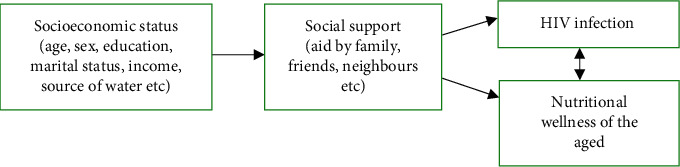
Model of HIV infection and nutritional wellness of the aged.

Thus, the specific link between aged persons with HIV infection and their nutritional wellness is missing out. In this study, we modelled that socioeconomic status, social support, HIV infection, and nutrition are closely related together. Therefore, socioeconomic status affects social support, and these two (socioeconomic status and social support) affect HIV infection and nutritional wellness. Finally, HIV infection affects nutritional wellness and vice versa (see Figure 1).

## 2. Materials and Methods

### 2.1. Data Source

This study was a secondary analysis of a sample survey data from 2013 South Africa-SAGE Well-Being of Older People Study (WOPS) Wave 2 [23]. SAGE WOPS surveys are designed by the World Health Organization and the Africa Centre for Health and Population Studies and implemented by the Africa Centre for Health and Population Studies. SAGE WOPS-South Africa provides a comprehensive dataset on the roles and health issues of older people (50 years and older) who have descendants who are infected or deceased due to HIV, or who have HIV themselves [23, 24]. More specifically, it describes the effects on physical and mental health, household income, and social situation, as well as the tasks and responsibilities of older people infected and/or affected by HIV. Further, the effects of the introduction of HIV treatment on the lives and well-being of people aged 50 and above were investigated [25].

SAGE WOPS survey compiles comparable longitudinal information on the health status, well-being, and functional status among aged persons either infected with HIV themselves or affected by HIV/AIDS in their families in South Africa. Besides, the survey also looked at the demographic parameters of categories of aged persons and HIV treatment, age, and nutritional status of respondents. Also, the sample for the survey was stratified into five groups [25]. Sample for Group 1 were the aged who are on HIV treatment for 1 year or more in 2010 at the time of Wave 1 of the project. Group 2 was made up of the aged who were not on HIV treatment or on treatment for 3 months or less in 2010 of Wave 1. The third Group included the aged who had adult (14–49 years) children in the household, who were HIV infected in 2010 of Wave 1. Group 4 comprised aged persons who had experienced an HIV-related death of an adult household member in 2010 of Wave 1. Lastly, Group 5 included the aged who were not on HIV treatment or were on treatment for 3 months or less in 2013 during Wave 2 [25].

There was oversampling of participants in Groups 2 and 5. A two-stage sampling process was adopted for the aged in Groups 1, 2, and 5. At stage one, all persons meeting the respective criteria for each group were identified from the Hlabisa treatment program. At stage two, 100 participants for each group who are also under surveillance were randomly selected. A total of 519 aged 50 years and over participated in the SAGE WOPS sample survey Wave 2. Therefore, the respondents (*n* = 440) who had complete data constituted the sample for this study. Respondents were classified into four categories of age brackets: the “younger old” (50–64), “young old” (65–74 years); the “old old” (75–84 years); and the “oldest old” (85 years and above) [23–25]. The data were deemed suitable for this study, because it is nationally representative.

### 2.2. Study Variables

#### 2.2.1. Dependent Variable

**Figure 2:**
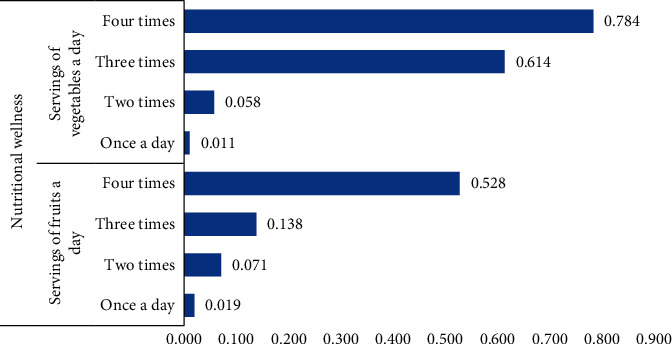
Factor loadings of nutritional wellness.

The outcome variable of interest focused on nutritional wellness. Nutritional wellness is used to refer exclusively to improvement of health in later life through a balance diet. The data covered 440 aged persons who were affected or infected by HIV. The response variable in the data included the following: How many servings of fruit do you eat on a typical day? How many servings of vegetables do you eat on a typical day? These were coded, 0 = no servings, 1 = one serving, 2 = two servings, 3 = three servings, 4 = four servings, and 5 = five-eight servings [11]. Dummies were created for each of the four nutritional variables. Principal Component Analysis was used to create an index for nutritional wellness. The factor loadings in Figure 2 show that the higher the number of times aged persons are served fruits and vegetables, the better their nutritional wellness. The response variable was then created into a binary, coded 0 = low nutritional wellness, 1 = high nutritional wellness.

#### 2.2.2. Independent Variable

The main independent variable was HIV status of aged persons. This was derived from (1) the SAGE WOPS group (the aged on HIV treatment for 1 year or more in 2010 [Wave 1], (2) the aged who were not on HIV treatment or on treatment for 3 months or less in 2010 [Wave 1], (3) the aged with adult (14–49 years) children in the household who were HIV infected in 2010 [Wave 1], (4) the aged who had experienced an HIV-related death of an adult household member in 2010 [Wave 1], and (5) the aged who were not on HIV treatment or were on treatment for 3 months or less in 2013 [Wave 2]). These were recoded into “(1,2,5) 1 = infected”, and “(3,4) 2 = noninfected”. Apart from the key independent variable (SAGE WOPS group), sex (coded 1 = male, 2 = female), age (recoded 1 = 50–64, 2 = 65–74, 3 = 75–84, 4 = 85+), marital status (recoded 1 = not married, 2 = married, 3 = divorced/separated, 4 = widowed), level of education (recoded 1 = no formal education, 2 = primary, 3 = secondary, 4 = tertiary), source of drinking water (recoded 0 = other source, 1 = treated tap water, 2 = rainwater, 3 = flowing river/stream, 4 = dam/stagnant water), household source of income (recoded 0 = no source of income, 1 = self-generated income, 2 = wages/salaries, 3 = government grants, 4 = rental property/retirement fund), and current financial situation (recoded 1 = much worse, 2 = about the same, 3 = better) were considered as covariates [26, 27]. The recoding of variables was done to suit the analysis.

### 2.3. Data Analysis

We conducted both descriptive and inferential analyses. At the descriptive level, we analysed the background characteristics of the aged (univariate). We also computed the proportion of the aged with high nutritional wellness by background characteristics (bivariate). Chi-square tests were used to investigate significant differences (*p* < 0.05). Afterwards, multilevel logistic regression techniques were used to examine high nutritional wellness and HIV status among the aged. Two-level binary logistic regression models were fitted. The first model examined the HIV status and nutritional wellness (Model I). The second model (Model II) was a complete model accounting for all the four important explanatory variables (Table 1). The output was reported as Odds Ratios (ORs) and Adjusted Odds Ratios (AORs) for Model I and Model II, respectively, at 95% confidence interval. The entire analysis was carried out using SPSS version 25 and R for Windows.

### 2.4. Ethical Approval

The South Africa-SAGE Well-Being of Older People Study (WOPS) Wave 2 was approved by the Ethics Review Committee and the World Health Organization, Geneva, Switzerland. Written informed consent was given by all individuals. Authors of this manuscript were not directly involved in the data collection processes but rather obtained access by requesting for the data.

### 2.5. Results and Discussion

Results from the univariate analysis show that there were more aged women (79.8%) than men (20.2%) in the Somkhele, South Africa. Young old (50–64) dominated (65.7%) in the ages, among the respondents. A higher percent of the aged were widows (39.5%) and had no formal education (46.1%). With respect to source of drinking water and household income, results in Table 2 show that more than half of the aged drank from treated tap water (62.5%) and received government grants (69.8%). Almost half of the respondents described their current financial situation as much worse (49.1%). Considering fruits and vegetable servings, results show that a higher percent (38.9%) had three servings of fruits and just one serving of vegetables (59.1%) in a typical day. Six in 10 aged persons were HIV infected (59.5%).

#### 2.5.1. Bivariate Analysis of Sampled Aged High Nutritional Wellness by Background Characteristics

Table 3 shows the results of sampled aged high nutritional wellness by background characteristics. Results show that HIV status, sex, age, education, source of drinking water, household source of income, and current financial situation had significant impact on nutritional wellness of the aged in Somkhele, South Africa. Most of the aged had a high nutritional wellness, but the percentage distribution of men was higher. Aged persons with HIV infection (76.7%) had nutritional wellness. When compared to the old old (75–84) and oldest old (85+), the younger old (50–64) and young-old (65–74) had high nutritional wellness (75.4% and 74.8%). The aged who were not married showed high nutritional wellness (77.6%) when compared to those who were separated/divorce (71.9%) and those who were married (72.0%), although differences in percentage distribution were not large enough to be statistically significant. Further results in Table 3 show that the aged without any formal education (77.8%) had high nutritional wellness compared to those with primary level of education (68.2%). Interestingly, the majority of the aged who drank from dam/stagnant water and with no source of household income had high nutritional wellness (81.0% and 97.7%), respectively. Notably, those with much worse financial situations experienced high nutritional wellness (89.8%).

#### 2.5.2. Regression Analysis of HIV Status and High Nutritional Wellness among the Aged

Table 1 presents regression analysis of HIV status and high nutritional wellness among the aged. The results in Table 1 show that the estimated AIC for Model 0 (null model) was 511.6. When the individual variable was introduced in Model I, the AIC was reduced by 1.1. The results show that the odds of having nutritional wellness were low for aged persons who were infected by HIV (OR = 0.74, 95% CI = 0.69, 1.26) when compared to those who were not infected. This did not change much after introducing the confounders.

The AIC reduced further to 64.1 after the confounders were added in Model II. The odds of having nutritional wellness reduced slightly but were still low among aged persons who were HIV infected (OR = 0.71, 95% CI = 0.61, 1.48) when compared with those who were not infected. Regarding ages of respondents, the odds of having nutritional wellness were 12.2 percent higher for young old (65–74 years) (OR = 1.22, 95% CI = 0.66, 2.27) compared to younger old (50–64 years). Further results show that aged persons with primary level of education were less likely to experience high nutritional wellness (OR = 0.53, 95% CI = 0.28, 0.99) when compared with those with no formal education. Compared to those who had no source of income, aged persons who received government grants were less likely to have high nutritional wellness (OR = 0.19, 95% CI = 0.03, 1.45). Meanwhile, the aged with better financial situation had highest odds of high nutritional wellness (OR = 6.15, 95% CI = 2.23, 16.93) as compared with those with much worse financial situation. Considering the confounders, age, level of education, household source of income, and financial situation were important in high nutritional wellness of the aged.

The variance terms presented in Table 1 are statistically significant at HIV status of respondents (individual level). Model I reveals that there are significant differences among aged persons with HIV status and high nutritional wellness. When the confounding factors were introduced into the model, the individual variance was reduced by an extremely small margin in percent. Meanwhile, the between-individual variance was still significant. This shows that the variables that were accounted for in the model clearly explain the between-individual variations in high nutritional wellness of the aged and their HIV status.

## 3. Discussion

Globally, HIV infection has significant impact on aged HIV infected persons in low- and middle-income countries like South Africa, and low nutritional wellness is a major contributing factor [28]. This study aimed to examine HIV status and nutritional wellness of aged persons in Somkhele, South Africa. We found that having HIV infection was associated with low nutritional wellness when compared with those who were not infected. Low nutritional wellness has many health implications for aged persons with HIV infection. Somarriba et al. [29] assert that aged HIV-infected patients have increased delayed immune response. This is because good nutritional wellness improves the quality life span and symptom management, supports the effectiveness of medications, and improves the patient's resistance to infections and other disease complications by altering immunity [29]. Thus, optimal nutrition is an important supplement in the clinical care of patients with HIV infection [29]. Meanwhile, adequate nutritional status supports immunity and physical performance [30]. Moreover, HIV is known to negatively impact the nutritional health of an individual in three underpinning ways including the fact that it alters the body's metabolism so that more energy, protein intake, and micronutrients are demanded and utilised [3]. In furtherance, individuals with HIV often eat less food due to loss of appetite, mouth or throat sores, pain and nausea, side effects of medication, or worsening household poverty and livelihood security, and finally, it impairs the absorption of nutrients consumed on account of diarrhea and vomiting, damaged intestinal cells, and other effects of opportunistic infections [3]. It could also mean that HIV-infected aged persons are living alone, which increases the chance of them not eating recurrently [9]. In this regard, correcting nutritional status becomes more difficult as infection progresses [30]. This is because of the widespread micronutrient deficiencies among HIV-infected people [30]. Our finding supports the inter-relationship between HIV status and nutritional wellness of aged persons as purported in our model.

Regarding ages of respondents, findings show that having nutritional wellness was 12.2 percent higher for young old (65–74 years) compared to younger old (50–64 years). Similarly, a study in Swaziland found that HIV prevalence rate was between 13% and 7% among young old (65–74 years) [11]. On the other hand, Gregório et al. [10] found HIV prevalence ranging between 5% and 13% among younger old (50–64 years) in Kenya and South Africa. Meanwhile, Han et al. [31] found that age is one of the best predictors and significantly associated with nutritional wellness. However, their study did not tell which of the distinct categories of ages that are more likely to be associated with nutritional wellness, and among aged HIV infected persons.

Further results show that aged persons with primary level of education were less likely to experience high nutritional wellness when compared with those with no formal education. This could imply that less-educated aged persons rely more upon different specific sources for their nutrition information than those who have attained a higher level of education [32].

Compared to those who had no source of income, we found that aged persons who received government grants were less likely to have high nutritional wellness. This presupposes that government grants might not be received regularly by aged persons. This corroborates with findings by Bekele et al. [33] who also discovered that low annual household income and difficulties in meeting housing-related expenses are independently associated with low nutritional wellness.

Also, the aged with better financial situation had highest odds of high nutritional wellness as compared with those with much worse financial situation. This confirms the finding by Bowman, [34] which shows that a smaller proportion of low-income aged persons ate less Adequate Intake or Estimated Average Intake levels for many micronutrients than those with high income. They ate less fruit, vegetables, milk, meat, poultry, and fish than the high-income aged. This highlights the fact that being HIV infected but having better financial situation can contribute positively to better nutritional status of aged persons with HIV infection. Thus, aged persons with low-income or worse financial situations face various health challenges, particularly related to poor mental health and nutritional and physical function [31, 35]. Considering the confounders, age, level of education, household source of income, and financial situation had statistically significant association with HIV status and nutritional wellness of the aged.

This study draws its conclusions from representative sample size of HIV-infected aged persons, also drawn from the use of a multistage sampling method in the selection of the respondents and the rigorous statistical analysis carried out. The questionnaires and methods of data collection have also been validated. However, due to the cross-sectional nature of the survey, we could not establish causality. We also used only variables with complete cases for our analysis, which has the potential of producing biased estimates.

## 4. Conclusion

This study suggests that age, education, source of drinking water, household source of income, and financial situation are important for nutritional wellness of aged persons who are HIV infected in Somkhele, South Africa. Moreover, having HIV infection is associated with low nutritional wellness. There is interrelationship between HIV status and nutritional wellness. Being a young old (65–74 years), receiving government grants, and with better financial situation has high nutritional wellness. On the other hand, aged persons with primary level of education have low nutritional wellness. Imperatively, stakeholders and agencies who have interest in the aged living with HIV must understand the socioeconomic status in relation to their nutritional wellness. The results from this study fill in literature gaps and are of significant importance to ageing policies, specifically in health and nutrition.

## Figures and Tables

**Table 1:** Regression analysis on high nutritional wellness among the aged.

Covariates	Model I	Model II
	OR [95% CI]	OR [95% CI]
HIV status		
Not infected	1.00	1.00
Infected	0.74 [0.96, 2.26]	0.71 [0.80, 2.48]

Confounding factors		
Individual level factors		
Age		
50–64	—	1.00
65–74	—	1.22 [0.66, 2.27]
75–84	—	0.81 [0.31, 2.07]
85+	—	0.09 [0.01, 0.73]^*∗*^

Level of education attained		
No formal education	—	1.00
Primary	—	0.53 [0.28, 0.99]^*∗*^
Secondary	—	0.71 [0.37, 1.37]
Tertiary	—	0.62 [0.10, 3.75]

Household source of income		
No source of income	—	1.00
Self-generated income	—	0.08 [0.01, 0.73]^*∗*^
Wages/salaries	—	0.09 [0.01, 0.73]^*∗*^
Govt grants	—	0.19 [0.03, 1.45]
From rental property/retirement fund	—	0.08 [0.00, 1.37]

Financial situation		
Much worse	—	1.00
About the same	—	1.6 [0.45, 2.35]
Better	—	6.15 [2.23, 16.93]^*∗∗∗*^

Variance of the random effects (SE)		
Individual level	1.69 *E*−07 [2 *E*−05]^*∗∗*^	0.1937 [2.10 *E*−02]^*∗∗∗*^
%∆ in random effect	−3 *E* + 03	−1 *E* + 06

Individual level		
Deviance	503.4	415.8
%∆ in deviance	0.0	0.2
AIC	510.5	446.4

^*∗∗∗*^ *p* < 0.001, ^*∗∗*^*p* < 0.01, and ^*∗*^*p* < 0.05; SE, standard error; 95% CI, 95 percent confidence interval; AIC, akaike information criterion.

**Table 2:** Background characteristics of sampled aged (*n* = 440).

Background characteristics	Sample size	Percent
Sex		
Male	89	20.2
Female	351	79.8

Age		
50–64 years	289	65.7
65–74 years	107	24.3
75–84 years	37	8.4
85+	7	1.6

Marital status		
Not married	116	26.4
Married	118	26.8
Separated/divorce	32	7.3
Widowed	174	39.5

Level of education		
No formal education	203	46.1
Primary	129	29.3
Secondary	98	22.3
Tertiary	10	2.3

Source of drinking water		
Other	2	0.5
Treated tap water	275	62.5
Rainwater	10	2.3
Flowing river/stream	27	6.1
Dam/stagnant water	126	28.6

Household source of income		
No source of income	43	9.8
Self-generated income	24	5.5
Wages/salaries	61	13.9
Govt grants	307	69.8
From rental property/retirement fund	5	1.1

Financial situation of household compared to 3 years ago		
Better	36	8.2
About the same	188	42.7
Much worse	216	49.1

Servings of fruits		
No servings of fruits	1	0.2
One	127	28.9
Two	171	38.9
Three	122	27.7
Four	15	3.4
5/8	4	0.9

Servings of vegetables		
No servings of vegetables	5	1.1
One	260	59.1
Two	135	30.7
Three	35	8.0
Four	5	1.1

HIV status		
Infected	262	59.5
Not infected	178	40.5

**Table 3:** Distribution of the sampled aged by background characteristics (*n* = 440).

Background characteristics	High nutritional wellness		
	%	95% CI	*p* value
Overall	73.6	—	—
HIV status	—	—	0.007
Infected	76.7	[0.71, 0.83]	—
Not infected	69.1	[0.61, 0.77]	—

Sex	—	—	0.023
Male	78.7	[0.70, 0.87]	—
Female	72.4	[0.68, 0.77]	—

Age	—	—	0.003
50–64 years	75.4	[0.70, 0.80]	—
65–74 years	74.8	[0.67, 0.83]	—
75–84 years	64.9	[0.50, 0.80]	—
85+	28.6	[−0.05, 0.62]	—

Marital status	—	—	0.074
Not married	77.6	[0.70, 0.85]	—
Married	72.0	[0.64, 0.80]	—
Separated/divorce	71.9	[0.56, 0.87]	—
Widowed	72.4	[0.66, 0.79]	—

Level of education	—	—	0.027
No formal education	77.8	[0.72, 0.84]	—
Primary	68.2	[0.60, 0.76]	—
Secondary	72.4	[0.64, 0.81]	—
Tertiary	70.0	[0.42, 0.98]	—

Source of drinking water	—	—	0.000
Other	50.0	[-0.19, 1.19]	—
Treated tap water	74.2	[0.69, 0.79]	—
Rainwater	50.0	[0.19, 0.81]	—
Flowing river/stream	44.4	[0.26, 0.63]	—
Dam/stagnant water	81.0	[0.74, 0.88]	—

Household source of income	—	—	0.000
No source of income	97.7	[0.93, 1.02]	—
Self-generated income	66.7	[0.48, 0.86]	—
Wages/salaries	49.2	[0.37, 0.62]	—
Govt grants	76.2	[0.62, 0.72]	—
From rental property/retirement fund	40.0	[−0.03, 0.83]	—

Financial situation of household compared to 3 years ago	—	—	0.000
Better	50.0	[0.34, 0.66]	—
About the same	59.6	[0.53, 0.67]	—
Much worse	89.8	[0.86, 0.94]	—

## Data Availability

The dataset can be accessed by request at https://apps.who.int/healthinfo/systems/surveydata/index.php.
